# Machine learning-based detection of acute psychosocial stress from body posture and movements

**DOI:** 10.1038/s41598-024-59043-1

**Published:** 2024-04-08

**Authors:** Robert Richer, Veronika Koch, Luca Abel, Felicitas Hauck, Miriam Kurz, Veronika Ringgold, Victoria Müller, Arne Küderle, Lena Schindler-Gmelch, Bjoern M. Eskofier, Nicolas Rohleder

**Affiliations:** 1 Machine Learning and Data Analytics Lab (MaD Lab), Department Artificial Intelligence in Biomedical Engineering (AIBE), Friedrich-Alexander-Universität Erlangen-Nürnberg (FAU), 91052 Erlangen, Germany; 2 Fraunhofer Institute for Integrated Circuits IIS, 91058 Erlangen, Germany; 3 Chair of Health Psychology, Friedrich-Alexander-Universität Erlangen-Nürnberg (FAU), 91052 Erlangen, Germany; 4 Chair of Clinical Psychology and Psychotherapy, Friedrich-Alexander-Universität Erlangen-Nürnberg (FAU), 91052 Erlangen, Germany; 5 Translational Digital Health Group, Institute of AI for Health, Helmholtz Zentrum München - German Research Center for Environmental Health, 85764 Neuherberg, Germany

**Keywords:** Health psychology, Stress, Cortisol, TSST, Motion capturing, Freezing, IMU, Machine learning, Human behaviour, Computer science, Scientific data, Biomedical engineering

## Abstract

Investigating acute stress responses is crucial to understanding the underlying mechanisms of stress. Current stress assessment methods include self-reports that can be biased and biomarkers that are often based on complex laboratory procedures. A promising additional modality for stress assessment might be the observation of body movements, which are affected by negative emotions and threatening situations. In this paper, we investigated the relationship between acute psychosocial stress induction and body posture and movements. We collected motion data from N = 59 individuals over two studies (*Pilot Study*: N = 20, *Main Study*: N = 39) using inertial measurement unit (IMU)-based motion capture suits. In both studies, individuals underwent the Trier Social Stress Test (TSST) and a stress-free control condition (friendly-TSST; f-TSST) in randomized order. Our results show that acute stress induction leads to a reproducible freezing behavior, characterized by less overall motion as well as more and longer periods of no movement. Based on these data, we trained machine learning pipelines to detect acute stress solely from movement information, achieving an accuracy of ${75.0 \pm 17.7}{\%}$ (*Pilot Study*) and ${73.4 \pm 7.7}{\%}$ (*Main Study*). This, for the first time, suggests that body posture and movements can be used to detect whether individuals are exposed to acute psychosocial stress. While more studies are needed to further validate our approach, we are convinced that motion information can be a valuable extension to the existing biomarkers and can help to obtain a more holistic picture of the human stress response. Our work is the first to systematically explore the use of full-body body posture and movement to gain novel insights into the human stress response and its effects on the body and mind.

## Introduction

Experiencing acute stress induces strong physiological reactions aimed at setting the human body into a state of alertness, thereby allowing it to rapidly adapt to new situations or challenges^1,2^. One particular form of stress we frequently encounter in our daily lives is psychosocial stress, which is caused by the interactions with of our social environment. It can arise from a wide range of sources, such as work, relationships, and daily life demands^3^. It activates the two major stress systems of the human body, the sympathetic nervous system (SNS) and the hypothalamic-pituitary-adrenal (HPA) axis^4^. When the body experiences stress, the faster SNS is activated, which initiates the “fight-or-flight” response. This leads to the release of hormones such as adrenaline and nor-adrenaline which prepare the body for physical action by increasing heart rate and blood pressure, among others. At the same time, the slower HPA axis is stimulated, which results in the release of the hormone cortisol from the adrenal glands. Cortisol helps to mobilize energy resources, modulates sympathetic effects, and plays an important role in regulating inflammation. Together, these changes enable the body to respond effectively to the stressor^2,4,5^.

The acute stress response is a normal and healthy reaction to challenging or threatening situations, and can even serve as a motivator to help us meet the demands of our environment^6^. However, it can also transition into chronic stress, ultimately leading to the known negative effects on a person’s physical and mental health, such as depression, anxiety, cardiovascular diseases, and diabetes^7^. Repeated acute stress can lead to allostatic load, which describes the “wear and tear” of stress systems, resulting in inadequate biological responses and making it more difficult to manage future stressors^1^. Thus, understanding the underlying physiological reactions to acute psychosocial stress can help to identify individuals at risk, and, potentially, prevent the transition from acute into chronic stress^8,9^.

Traditionally, acute stress is induced in the laboratory. Using standardized procedures the resulting stress response is measured by self-reports to assess subjective experiences of stress, and by neuroendocrine biomarkers, such as cortisol^10^ and salivary alpha-amylase^11^, to assess SNS and HPA axis reactivity, respectively. Additionally, SNS reactivity can be characterized by measuring electrophysiological signals, such as electrodermal activity (EDA) or electrocardiography (ECG), from which heart rate (HR) and heart rate variability (HRV) can be derived^12^. To measure the body’s inflammatory response to stress, inflammatory markers, such as C-reactive protein (CRP) and Interleukin-6 (IL-6), are typically measured^13^.

However, these markers suffer from various drawbacks which can limit the further development of biopsychological research. For instance, self-reports can be administered quickly and easily and can help to provide direct information about an individual’s subjective experiences and perceptions during acute stress. However, they can be subject to bias and variability since individuals might not be able to accurately report their experiences since they have different perceptions and experiences of stress, or might be influenced by factors such as social desirability^14^. In contrast, HR(V) is simple to measure, even contactless^15^, but not very sensitive to stress since similar physiological responses are also caused by other factors, such as physical activity, caffeine intake, or general arousal^16^. Lastly, neuroendocrine markers allow us to quantify the biological stress response more objectively but involve a high effort for both researchers and study participants since they are based on complex, often invasive, laboratory procedures^17^. Furthermore, they only allow the measurement of the *response* after, but not an individual’s behavior *during* acute stress.

A promising extension to the established biomarkers might be the observation of body posture and movements. They can yield valuable insights about an individual’s inner state and, thus, can potentially be used for stress detection^18–21^. Research has already identified certain movement patterns typical for individuals under stress. For instance, Atkinson et al. showed that negative emotions, such as shame, can be characterized by three types of movements: Dropping the head, bringing the hands to the face, and crossing the arms in front of the body^22^. In comparison, an elevated head can signal positive emotions, such as pride^23^. These findings motivate the potential for stress detection from body posture as both emotions are causally related to the cortisol stress response^24^. Another common behavioral response is a significant reduction in body movements, also referred to as *freezing*^25^. For instance, Doumas et al.^26^ reported that exposure to such a threat leads to a reduction in postural sway and fewer body movements. Similar findings were also observed when individuals were confronted with angry faces or affective films^25,27^. Furthermore, Zito et al.^18^ suggested that freezing is linked to the biological stress response since patients diagnosed with functional movement disorders (FMD), who are known to show abnormal stress responses, also showed impaired freezing responses during stress exposure. Pisanski et al.^28^ showed that exposure to acute psychosocial stress leads to a significant reduction in hand movements, measured using a polygraph, compared to a pre-stress baseline. They concluded that the act of deception during the Trier Social Stress Test^29^, the gold standard for acute psychosocial stress induction^24^, poses a situation of acute social threat that can also be observed among people while they are lying^19^. While the presented approaches highlight the relationship between bodily movements and stress or negative emotions, their analyses were mostly restricted to single body parts and did not systematically assess and analyze whole-body movements.

Addressing this gap, van der Zee et al. recorded full-body movements to detect deceit by computing and comparing the absolute body movement of individuals. Their approach yielded an accuracy of 82.2% for distinguishing whether interviewees were lying or telling the truth when combining full-body motion measurements with information from individual limb movements^20^. However, they experienced that individuals who were lying showed more body movements compared to people who were telling the truth. Even though they did not induce stress in their study their results motivate further research since they emphasize the link between body movements and situations that are perceived as negative and threatening. Similar results to the ones presented by van der Zee et al. were obtained by Arnrich et al.^21^ who attempted to discriminate stress from cognitive load by measuring the pressure distribution in a chair with integrated sensors. Their results suggested that seated individuals under stress showed more body movements compared to a control condition in which mild cognitive load was induced. Using this information, they were able to distinguish stress from cognitive load with an accuracy of 73.8%.

Despite this link, the effect of acute psychosocial stress on body posture and movement and the use of body posture as a marker for stress measurement has remained largely unexplored. One possible reason for this lack of research is the difficulty in accurately measuring and quantifying body posture and movement without encumbering the individual. The gold standard for measuring full-body movements is marker-based optical motion capturing (OMC), where cameras are utilized to accurately track human movement with the help of reflective markers placed on the human body. Afterwards, the 3-d marker positions are computed using triangulation methods^30^. However, since these markers need to be attached to the skin to achieve high data quality, this often requires study participants to be only lightly dressed which might make them feel uncomfortable, especially during situations of social-evaluative threat. Additionally, these systems require specialized OMC laboratories, which might not be easily accessible for (stress) researchers. Finally, the awareness of being observed can influence the behavior of individuals which can lead to measurement bias for stress assessment^31^. An alternative could be the use of inertial measurement unit (IMU)-based motion capturing approaches. Even though participants can still be aware of being observed, these systems are less intrusive since participants can remain fully dressed. Despite being less accurate than marker-based OMC, IMU-based systems benefit from lower costs, higher portability, ease of use, and independence from external references^32,33^, making them potentially better suited for use in acute stress scenarios.

To address the gap of motion assessment in biopsychological research, we investigate the effect of acute psychosocial stress on body posture and movements by measuring the full-body motion of individuals in the Trier Social Stress Test (TSST)^29^, the laboratory stress induction gold standard^24^, and the friendly-TSST (f-TSST), a control condition that is as similar as possible to the TSST but does not activate the HPA axis^34^. To the best of our knowledge, our work is the first to systematically investigate this effect in a *within* setting with an adequate control condition, giving us the unique opportunity to assess the effect of social-evaluative threat, as induced in the TSST, on body posture and movements of individuals. We hypothesize that acute psychosocial stress leads to a significant reduction in body movements compared to the stress-free control condition. Furthermore, we explore the potential of using body posture and movement information for the machine learning-based detection of acute psychosocial stress. To this end, we extract features from the measured body posture and movement data and train machine learning models to classify acute psychosocial stress. With our work, we aim to lay the foundation for further research in investigating body posture and movement information as behavioral markers during stress and explore its potential as an extension to the established biopsychological markers.

## Methods

### Data acquisition

#### Recruiting and screening

To measure body posture and movements during acute stress, we designed and conducted two studies during which each individual was exposed to the TSST and the f-TSST on two consecutive afternoons. The first study served as a *Pilot Study* to test the feasibility of the experimental setup and to refine the measurement techniques and protocols. This initial study allowed us to gather preliminary data on how body posture and movements change under stress and to identify any technical or procedural issues that needed addressing. Following the insights gained from our *Pilot Study*, we designed our *Main Study* and preregistered it at Open Science Foundation (OSF) (https://osf.io/rewhc).

For both studies, we recruited young, healthy individuals through advertisements in psychology and engineering lectures at Friedrich-Alexander-Universität Erlangen-Nürnberg (FAU), as well as via social media and flyers. Our studies were approved by the ethics committee of FAU (protocol #493_20 B) and were performed in accordance with the Declaration of Helsinki. Before testing, we obtained written informed consent from all participants.

Potential study participants were first asked to complete a screening questionnaire assessing their eligibility for participation. Exclusion criteria were defined according to previous studies^35,36^ and recommended guidelines for measuring HPA axis activity^17,37^ and included:

1. Age lower than 18years or higher than 40years,
2. Body mass index (BMI) lower than ${18}\textrm{kgm}^{-2}$ or higher than ${30}\textrm{kgm}^{-2}$,
3. Presence of physical or mental diseases,
4. Medication intake (such as beta-blockers, glucocorticoids, anti-depressants),
5. Consumption of cigarettes or other drugs,
6. Self-reported depression assessed by the “Allgemeine Depressionsskala” (ADS-L)^38^, the German version of the “Center for Epidemiological Studies Depression Scale” (CES-D)^39^ (using a cut-off of $n>22$), and
7. Previous experience with stress tests.

In our *Main Study*, we additionally excluded female participants who were using hormonal contraceptives and were, according to self-report, not in the luteal phase of their menstrual cycles at the time of measurement because of associations between the use of hormonal contraceptives, menstrual cycle phase, and hormonal stress responses^40^.

In total, we recruited $N=21$ participants ($N=18$ women), aged ${22.6 \pm 4.0}$ years, BMI ${21.9 \pm 2.4}\textrm{kgm}^{-2}$ (M ± SD) for the *Pilot Study* between January and March 2021 and $N=41$ participants ($N=18$ women), ${24.0 \pm 3.5}$ years, BMI ${22.1 \pm 2.0}\textrm{kgm}^{-2}$, for the *Main Study* between March 2023 and May 2023. Participants were invited into the laboratory where the Trier Social Stress Test (TSST)^29^ and the friendly-TSST (f-TSST)^34^ were conducted in a within-design setting in randomized order on two consecutive days. To minimize the impact of circadian cortisol variations, we recorded participants between 1:00 p.m. and 9:00 p.m. and both conditions were performed at similar times of the day^41^. In advance, participants were further instructed to get up at least three hours before the study, to refrain from the consumption of alcohol on the preceding day and the day of the study, to not consume any food the hour before, and to avoid vigorous physical activity at least an hour before the study.

#### Procedure – *Pilot study*

After participants had arrived at the laboratory and completed the informed consent (only on Day 1), the experimenter ensured the participants adhered to the instructions before the study. Afterwards, the first (S0) of a total of six saliva samples per day was collected to assess HPA axis activity from salivary cortisol concentrations. Participants were instructed to move the polystyrol swabs of the Salivettes (Sarstedt AG & Co. KG, Nümbrecht, Germany) around in the mouth in a circular motion for 2min without chewing. S0 was assessed $t = -20\,\text {min}$ relative to the start of the (f-)TSST and served as a baseline to detect, and possibly exclude, individuals with high baseline cortisol levels. Afterwards, participants were asked to fill out a set of state questionnaires, including the state scale of the “State and Trait Anxiety Inventory” (STAI)^42^ to assess self-reported states levels of anxiety and the “Positive and Negative Affect Schedule” (PANAS)^43^ to measure positive and negative affect before the (f-)TSST, respectively.

**Figure 1:**
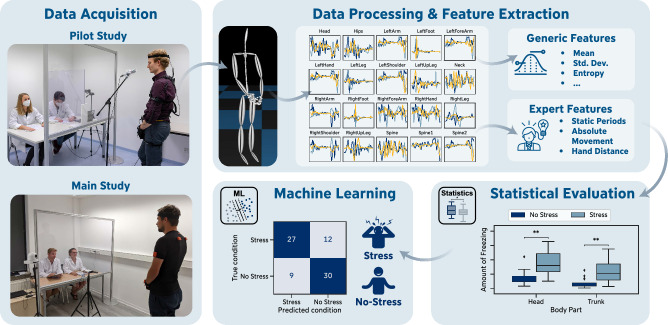
Overview of the key elements of this paper consisting of data acquisition during stress induction, data processing & feature extraction, statistical evaluation, and machine learning-based stress detection.

Following the questionnaires, the participants were equipped with the IMU-based motion capture suit (Perception Neuron, Noitom Ltd., Beijing, China). The system consists of 31 sensor nodes (“neurons”) and allows full-body motion capture at a sampling rate of 58.8 Hz. Each neuron comprises a 9-axis IMU sensor (accelerometer, gyroscope, and magnetometer). The neurons are attached to the body via straps and connected via cables. During data recording, the motion capture suit was connected via USB to a computer running the data recording software (Axis Neuron). A person wearing the motion capture suit during the TSST is visualized in Fig. 1.

Before starting the measurement, the experimenter calibrated the motion capture system by acquiring relevant body measures (height, shoulder width, upper arm width, lower arm width, palm width, and hip width) of each participant and creating personalized body size models in the Axis Neuron software. Following, participants were asked to perform four defined calibration poses:

1. *Steady pose*: sitting on a chair steadily while resting the hands on a table,
2. *A-pose*: standing straight with parallel feet, arms pointing downward, and palms facing towards the body,
3. *T-pose*: standing straight with parallel feet, arms extended straight out to the sides, palms facing downward, and fingers extended, as well as
4. *S-pose*: performing a half squat with parallel feet, arms extended straight out to the front, and palms facing downwards.

In case the Axis Neuron software reported bad calibration results, participants were asked to repeat the calibration procedure. Afterwards, the experimenter guided the participants to a different room where the (f-)TSST was conducted. Immediately before, the second saliva sample (S1, $t = -1\,\text {min}$) was collected.

The TSST was performed according to the protocol originally proposed by Kirschbaum et al.^29^. After the experimenter entered the room with the participants wearing the motion capture suit, they were introduced to a panel consisting of one female and one male experimenter wearing lab coats. In the room, the experimenter explained to the participants that they should imagine applying for their dream job and that the panel would be responsible for deciding whether they would get this job. Additionally, the participants were told that the panel was already aware of their professional qualifications and that they should, thus, only speak about their personality as the panel was trained in observing behavior and in the analysis of non-linguistic and body language signals. Once the instructions were explained, the study experimenter left the room and the TSST began.

In total, the TSST consists of three phases: *Preparation*, *Interview*, and *Mental Arithmetic*, each lasting for 5min. An important aspect of reliable stress induction is that participants remained unaware that the panel was specifically instructed to be completely neutral, show as little emotion as possible, and not engage in any interaction throughout the whole procedure. Additionally, only the panel member of the opposite gender is supposed to talk to the participant (also referred to as the *active* panel member), while the same-gender panel member (*passive*) remains silent.

The TSST started with the *Preparation* phase where participants were given time to prepare a talk about themselves for the upcoming interview while sitting at a desk. Then, they were asked to fill out the Primary Appraisal Secondary Appraisal (PASA)^44^, a questionnaire designed to assess cognitive appraisal processes in a stressful situation. After the Preparation, the *Interview* started. For that, the active panel member asked the participants to arise from the chair and position themselves in front of the panel where the passive panel member turned on a camera and microphone, both supposed to create higher levels of social evaluation. Afterwards, participants were asked to clap their hands to synchronize motion capture and video data for later analysis and start with their talk. They were expected to speak freely as long as possible. The active panel member only intervened when participants did not talk about their personalities, when they were not keeping eye contact with the panel, or when they remained silent for more than 20s. In the last phase, the participants were presented with a surprise *Mental Arithmetic* task where they were instructed to count backward from 2043 in steps of 17. In cases of mistakes, the active panel member intervened and the participants had to restart from 2043. After the end of the Mental Arithmetic phase, the motion capture recording was stopped and participants were dismissed.

In the *Pilot Study*, we conducted the f-TSST according to the protocol originally proposed by Wiemers et al. as a control condition for the TSST^34^. It was designed to be as similar as possible to the TSST with similar structure and cognitive demands but social-evaluative elements are removed. Thus, the panel members did not wear lab coats and were instructed to be friendly, supportive, and actively engage in conversations with the participants about their CVs and career aspirations. Due to the nature of the study procedure, the f-TSST can still induce physiological arousal, leading to by an increase in SNS activity and the resulting changes in heart rate (variability) and alpha-amylase^34^. However, due to the removal of social-evaluative elements, the f-TSST has been shown to not activate the HPA axis^34,45,46^. For that reason, we consider the f-TSST as *not stressful* in the sense that is does not induce acute psychosocial stress. The f-TSST also started with a 5min *Preparation* phase, followed by a 10min *Interview* phase. In comparison to the TSST, the original f-TSST protocol did not contain a *Mental Arithmetic* phase.

**Figure 2:**
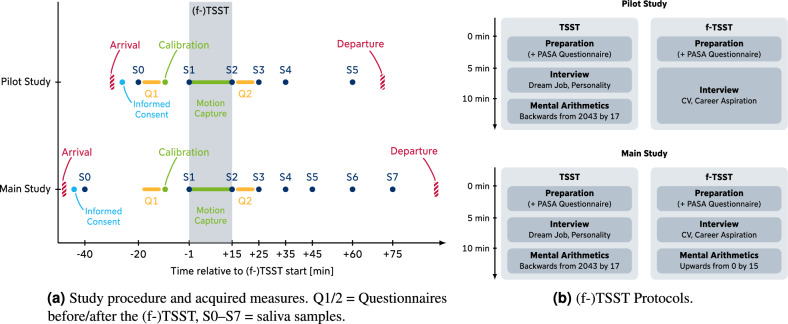
Visualization of study procedure (left) and (f-)TSST protocols (right) for *Pilot Study* and *Main Study*, respectively.

After the (f-)TSST, the participants were welcomed by the experimenter and guided into the previous room where the third saliva sample (S2, $t = +15\,\text {min}$ relative to (f-)TSST start) was collected. After taking off the motion capture suit, they were asked to complete the same questionnaires (STAI and PANAS, respectively) to assess psychological state variables after the (f-)TSST. In between, saliva samples S3-S5 were collected at time points $t = +25\,\text {min}, +35\,\text {min}, \text {and} +60\,\text {min}$, respectively. After the collection of S5, data collection finished and the participants were discharged from the laboratory and reminded to return for the second part of the experiment on the next day (Day 1) or debriefed about the mechanisms of the (f-)TSST (Day 2) and asked to sign a non-disclosure agreement (Fig. 2).

All collected saliva samples were stored at – 18 $^\circ$C for later analysis in the laboratory. Data from the motion capture suit were exported from the Axis Neuron software as .bvh and .calc files for later processing.

#### Procedure – *Main study*

The *Main Study* was conducted similarly to the *Pilot Study* to ensure comparability in the best possible way. However, based on insights from the *Pilot Study*, which are described in more detail the previous Section, we made some modifications to enhance the study design and methodology. We will outline these modifications in the following.

In the *Main Study*, we collected a total of eight saliva samples (S0-S7) at the time points $t = -40$, $-1$ $+15$, $+25$, $+35$, $+45$, $+60$, $+75\,\text {min}$ relative to (f-)TSST start, respectively. After S0, participants were provided with 200ml of grape juice (or 200mL of sugar water for participants with fructose intolerance) to minimize confounding effects due to interindividual differences in energy availability^47^.

Instead of the Perception Neuron motion capture system from the *Pilot Study*, which experienced considerable sensor drifts in position and rotation (Sect. Data processing), we used the Xsens MVN Awinda motion capture system (Movella, Henderson, NV, USA). It consists of 17 IMU sensor nodes attached to the body with velcro straps, allowing the full-body motion capture with a sampling rate of 60Hz. Similar to the Perception Neuron suit, the Xsens system was calibrated by, first, obtaining relevant body measures (body height, shoe length, shoulder height and width, wrist, elbow, and arm span, hip height and width, knee and ankle height) from each participant and, second, performing a calibration procedure. It consisted of maintaining a static standing pose (*Neutral pose*), followed by walking back and forth for about ten seconds (*Free walking*). The calibration procedure was repeated in case of bad calibration results reported by the software.

The TSST and f-TSST were conducted similarly as in the *Pilot Study*. However, we adapted the protocol of the f-TSST for better comparability to the TSST. Since the original f-TSST protocol did not contain a *Mental Arithmetics* phase, we shortened the *Interview* phase of the f-TSST from ten minutes to five minutes. We then added the *Mental Arithmetics* phase from the placebo-TSST^48^, another stress-free control condition to the TSST, which consists of counting upwards in steps of 15 starting at 0. If a mistake was made, the panel made participants aware of the mistake in a friendly way and asked them to continue from the last correct number. A comparison of the two study procedures is displayed in Fig. 2.

After finishing the (f-)TSST, we exported data from the Xsens system as .mvnx files for later processing.

### Measures

To verify successful acute psychosocial stress induction, we acquired the same biopsychological stress markers as in previous TSST studies^37^. We analyzed the saliva samples in the laboratory, where we first centrifuged all samples at 2000*g* and 20 $^\circ$C for 5min and then determined salivary cortisol concentrations in duplicate using a chemiluminescence immunoassay (CLIA, IBL, Hamburg, Germany) as described in more detail in previous publications^36^. The first saliva sample (S0) was disregarded from further analysis as it was only recorded for baseline comparison and the potential exclusion of study participants. Thus, we computed the area under the curve with respect to ground $AUC_G$ from S1–S5 (*Pilot Study*) and from S1–S7 (*Main Study*) as a measure for the total amount of secreted cortisol over time according to Pruessner et al.^49^. We additionally computed the maximum cortisol increase $\Delta c_{max}$ as a measure for the (f-)TSST-induced cortisol increase. The emotional stress response was assessed by computing the *Negative Affect (NA)* dimension from the PANAS and the *State Anxiety* dimension from the STAI questionnaire, respectively.

In addition to the established stress markers, we recorded body posture and movement during the (f-)TSST using full-body motion capture from the two motion capture systems. The Perception Neuron system (*Pilot Study*) yields data from a total of 21 joints (in the following also referred to as *body parts*) with five channels per body part: position in local coordinate system (pos), rotation in local coordinate system (rot), velocity (vel), angular velocity (ang_vel), and acceleration (acc). The Xsens system (*Main Study*) yields information from 23 body segments with 6 different channels: position and orientation in global coordinate system (pos_glo and ori_glo), velocity (vel), acceleration (acc), angular velocity (ang_vel), angular acceleration (ang_acc). Additionally, the joint angles (rot) from 22 joints are available.

**Figure 3:**
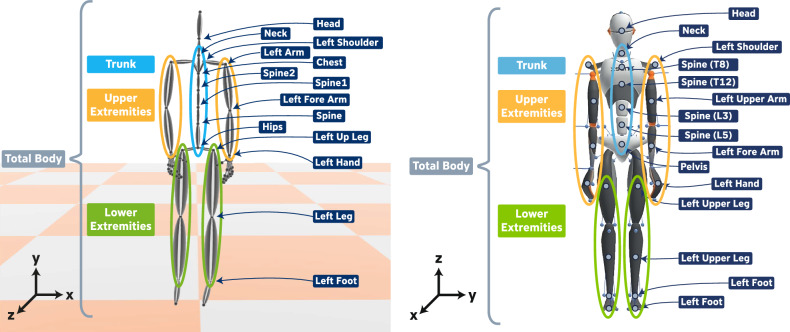
Body part (group) and coordinate definitions for the Perception Neuron (left) and Xsens (right) system.

For both systems, we additionally defined four body part groups (*Upper Extremities*, *Lower Extremities*, *Trunk*, and *Total Body*) to aggregate movements of one specific body region (Fig. 3).

### Data processing

The acquired data served as input for our data processing pipeline in which we extracted relevant features to characterize body posture and movement, aiming to distinguish acute stress from stress-free control conditions. These features serve as input for statistical analyses and the training and evaluation of different machine learning pipelines.

As the motion capture data from the Perception Neuron system (*Pilot Study*) were suffering from sensor drifts in position and rotation, we first filtered the data before further processing. Afterwards, we computed the global position and rotation (pos_glo and rot_glo, respectively). This preprocessing procedure is described in detail in the Supplementary Materials.

#### Feature extraction

To characterize the effect of acute psychosocial stress on body posture and movements, we selected a set of features and computed them on the given motion data. The features can be categorized into *generic* features that describe statistical properties and signal characteristics, thus not requiring any domain knowledge, and *expert* features that are based on previous work and behavioral observations of participants during the (f-)TSST, respectively.

We computed *generic features* on selected channels (vel, ang_vel, and rot for the *Pilot Study*; acc, vel, ang_vel, and rot for the *Main Study*) and selected body parts or body part groups. For each body part group, we determined the generic features by averaging the features computed of individual body parts contained in the respective group. We used information from single axes as well as from the L2-norm of the 3-d vectors for selected body parts for generic feature computation. An overview of the computed generic features is given in Table 1. Tables S1.1 and S1.2 list which features were computed for which body parts and channels for the *Pilot Study* and *Main Study*, respectively.

**Table 1:** Overview of generic features and their abbreviations.

Feature name	Abbreviation
Mean	mean
Standard deviation	std
Coefficient of variation	cov
Maximum value	max_val
Number of crossings through signal’s mean value	m_cross
Number of zero crossings	z_cross
Entropy	entropy
Absolute energy	abs_energy
Fast Fourier transform (FFT) spectrum centroid	fft_centroid
FFT spectrum variance	fft_var
FFT spectrum skewness	fft_skew
FFT spectrum kurtosis	fft_kurt

In addition to the generic features, we defined a selection of *expert features*. Similarly to van der Zee et al.^20^, we computed the *Absolute Movement*, which was first proposed by Poppe et al.^50^, of selected body part (groups) based on the global position (pos_glo) (Equation 1), where *N* denotes the number of motion capture frames, *J* denotes the set of body parts within the respective body part group, and *x* denotes the absolute position in 3-d:

1 $$\begin{aligned} \texttt {abs\_mov} = \frac{1}{(N-1)} \cdot \sum _{t=1}^{N}\left( \sum _{j \in J} \Vert x_{j,t} - x_{j,t-1}\Vert _{2} \right) . \end{aligned}$$

To quantify the expected freezing behavior during acute stress, we determined *Static Periods* in the vel and ang_vel channels of selected body part (groups) by windowing the signal and then computing the variance of the L2-norm of the 3-d vectors within the windows, similar to previous work^51^. We considered windows with a variance below a defined threshold $\theta _{sp}$ as *static* and merged consecutive static windows to longer static periods. For data from the Perception Neuron system (*Pilot Study*), we used a window length of 0.5s with an overlap of 50% and set $\theta _{sp}$ to ${1.0. 10^{-4} \textrm{m}^{2} \textrm{s}^{-2}}$ for vel and to $8.0. 10^{-4} \textrm{rad}^{2} \textrm{s}^{-2}$ for ang_vel. For data from the Xsens system (*Main Study*), we used a window length of 0.5s with an overlap of 50% and set $\theta _{sp}$ to $5.0. 10^{-5} \textrm{m}^{2} \textrm{s}^{-2}$ for vel and to $5.0\,^{{\circ }2} \textrm{s}^{-2}$ for ang_vel. All values were determined based on pre-studies with simulated freezing periods.

From the static periods, we then computed the start and end times and considered these as *freezing* periods. For body part groups, we determined freezing periods as the intersection of the freezing periods of all individual body parts. Afterwards, we computed the following metrics to characterize the freezing periods: The number of periods per minute, the ratio of total freezing duration to the total duration (in %), the duration of the longest freezing period (in s), the average duration of freezing periods (in s), and the standard deviation of freezing period durations (in s). A full list of expert features computed for the *Pilot Study* and *Main Study* is given in the Supplementary Tables S1.3 and S1.4.

In total, we computed 455 features comprising 390 generic and 65 expert features for the *Pilot Study* and 607 features comprising 509 generic and 98 expert features for the *Main Study*. We removed features that produced invalid results for any input data as well as features that were constant throughout all inputs. This cleaning process resulted in 427 features (365 generic and 62 expert features) used for later analysis for the *Pilot Study* and 587 features (509 generic and 78 expert features) for the *Main Study*.

### Availability of data and code

All (raw) data recorded during the experiment are available on OSF^52,53^. The Python libraries containing the source code for data processing, feature extraction, and reproducing all experiment results, figures, and tables are available on GitHub^54^.

### Evaluation

#### Exclusion criteria

For the evaluation of our studies, we defined the following exclusion criteria: (1) individuals with missing cortisol, self-report, or motion capture data, as well as (2) individuals with elevated baseline cortisol (S0) levels that are higher than three standard deviations from the average S0 cortisol concentration of the study population. Participants who met any of these criteria were excluded from all further analyses.

#### Statistical analyses

Before examining whether movement information can be used for stress characterization, we first analyzed whether the TSST conducted in our studies successfully induced acute psychosocial stress. Thus, we statistically compared the changes in cortisol levels and self-reports as a response to the (f-)TSST. Afterwards, we performed statistical analyses on the extracted motion features to assess whether the influence of acute psychosocial stress significantly changed body posture and movements.

As testing for normal distribution using the Shapiro-Wilk test revealed that some features violated this criterion, we performed non-parametric Wilcoxon signed-rank tests on all features with *condition* as between-variable since all participants were exposed to both conditions (TSST and f-TSST). We set a significance level of $$\alpha = 0.05$$ and reported effect sizes as Hedge’s *g*. To correct for the multiple comparisons problem, we applied Bonferroni corrections over all tests implemented for the same research question. We performed the same statistical analysis procedure for data from both studies and reported all p-values after Bonferroni correction. In all Figures and Tables, we used the following notation to indicate statistical significance: $$^{*} p< 0.05, ^{**} p< 0.01, ^{***} p < 0.001$$.

#### Classification experiments

We conducted a series of classification experiments to determine whether acute psychosocial stress can be detected from body posture and movements. Since we conducted the f-TSST as proposed in the original protocol^34^, a *Mental Arithmetic* phase was missing in the f-TSST of the *Pilot Study*. Thus, the study protocol is imbalanced which could bias the extracted features and the classification results. To examine this effect, we performed our classification experiments using two different motion feature sets: (1) extracted over the complete (f-)TSST procedure, and (2) extracted over the *Interview* phase of the TSST, and the first 5min of the f-TSST *Interview* phase, respectively. In contrast, the study protocol of our *Main Study* is more balanced since we added a *Mental Arithmetics* phase to the f-TSST protocol. Thus, we used motion features extracted over the complete (f-)TSST protocol as well as extracted over the *Interview* and *Mental Arithmetics* phases for the classification experiments of the *Main Study*, respectively.

As ground truth labels for all experiments, we used the condition in which the data were collected (TSST or f-TSST), assuming that individuals were stressed or not stressed in the respective condition. We compared different combinations of preprocessing, feature selection, and classification algorithms to determine the best pipeline with respect to classification performance. For preprocessing, we first removed all features with zero variance. Afterwards, we either scaled all features to a range of [0, 1] (Min-Max Scaler) or to a distribution with a mean value of zero and unit variance (Standard Scaler). For feature selection, we used Select-k-Best (SkB, based on the ANOVA F-value), Recursive Feature Elimination (RFE) using a linear Support Vector Machine (SVM) as base classifier, or Select From Model (SFM) which uses a Random Forest classifier with $n=100$ trees as meta-transformer to select features based on their feature importance scores. Lastly, we compared different classification algorithms: Naïve Bayes (NB), k-Nearest-Neighbors (kNN), Decision Tree (DT), Support Vector Machine with linear (SVM-lin), radial basis function (SVM-rbf) or polynomial kernel (SVM-poly), Random Forest (RF), Multi-Layer Perception (MLP), and AdaBoost (Ada).

We evaluated all different pipeline combinations using five-fold cross-validation (CV). Within each CV fold of the model evaluation, we used another five-fold CV for feature scaling and hyperparameter optimization of the feature selection and classification steps (also referred to as *nested CV*, as suggested by Vabalas et al.^55^). We used a randomized search with 40,000 iterations for the RF classifier and grid-search for all other classifiers. The ranges of the different hyperparameters are listed in Supplementary Table S2.

To avoid possible train-test leaks, we ensured that data from the same participant (TSST and f-TSST) were either present in the training or the test set for both the hyperparameter search CV and the model evaluation CV. As the target metric for hyperparameter optimization and model evaluation, we used the accuracy, defined as the number of correct classifications divided by all classifications. Afterwards, we retrained the pipelines with the hyperparameters that yielded the highest accuracy on all input data of the hyperparameter search CV to evaluate the different classification pipelines. We then performed predictions on the test set of the model evaluation CV, which the classifier has not seen yet. Based on the prediction results we derived confusion matrices for each classification pipeline combination and computed the classification metrics accuracy, precision, and F1-score over all folds of the model evaluation CV. We applied the same approach for on from both studies independently and performed all evaluation experiments using the Python libraries BioPsyKit (v0.10.2)^56^, tpcp (v0.13.0)^57^, pingouin (v0.5.4)^58^, and scikit-learn (v1.2.2)^59^.

## Results

Based on our exclusion criteria, we excluded one participant from the *Main Study* and two participants from the *Pilot Study*, both due to errors in the motion capture data recording leading to data loss. This results in $N=20$ (18 female, 2 male) and $N=39$ (18 female, 21 male) remaining participants for the subsequent statistical analyses and classification experiments. We will first present the results from our *Pilot Study*, which also provide the rationales for our subsequent *Main Study* and the actions we took to improve the experimental protocol, specifically the recruiting, the choice of motion capture system, and the protocol of the f-TSST. Afterwards, we will present the results from the *Main Study*.

### Pilot study

#### Biopsychological response to the (f-)TSST

**Figure 4:**
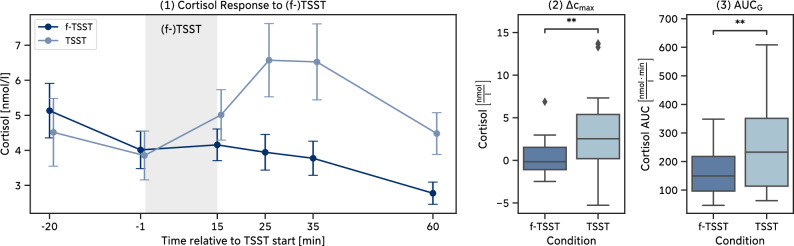
Pilot study: Cortisol responses to the (f-)TSST. (**1**) Mean ± standard error of cortisol samples over all participants within one condition, (**2**) maximum cortisol increase $\Delta c_{max}$, (**3**) area under the cortisol curve $\text {AUC}_{G}$.

Exposure to the TSST induced a significant cortisol increase in the participants of our *Pilot Study*, $W = 37.0, p = 0.019, g = 0.756$. On average, participants reached their peak in cortisol levels between 10min (*S3*) and 20min (*S4*) after the end of the TSST and started to recover afterwards. In contrast, the average cortisol levels after the f-TSST did not increase significantly, $W = 100.0, p > 0.999, g = 0.067$, and even continuously decreased over time (Fig. 4-1). In direct comparison between TSST and f-TSST, the TSST led to a significantly higher cortisol increase $\Delta c_{max}$, $W = 26.0, p = 0.004, g = 0.746$ (Fig. 4-2), and higher amounts of overall cortisol, characterized by $\text {AUC}_{G}$, $W = 31.0, p = 0.008, g = 0.709$ (Fig. 4-3).

Similarly, state anxiety and negative affect both significantly *increased* after the TSST (Anxiety: $W = 9.5, p = 0.001, g = 0.974$, Negative Affect: $W = 8.0, p = 0.003, g = 1.276$) while they significantly *decreased* after the f-TSST (Anxiety: $W = 23.0, p = 0.024, g = -0.554$, Negative Affect: $W = 9.0, p = 0.023, g = -0.636$). Direct comparison of both conditions showed that self-reported stress responses, measured as the difference of questionnaire scores after and before the (f-)TSST were significantly higher in TSST compared to the f-TSST (Anxiety: $W = 3.0, p < 0.001, g = 1.618$, Negative Affect: $W = 8.5, p = 0.001, g = 1.696$).

#### Effect of acute psychosocial stress on body posture and movement

After statistical testing and Bonferroni correction, 57 (14 generic, 43 expert) out of 447 total features computed from the *Pilot Study* showed significant differences between TSST and f-TSST (Supplementary Information S3).

**Figure 5:**
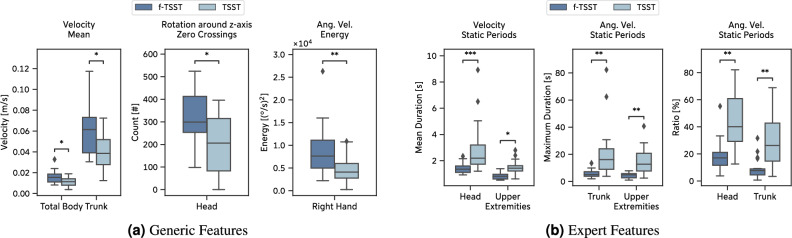
Pilot study: Selection of generic and expert features extracted from body posture and movements during the (f-)TSST.

The extracted features consistently support the assumption that acute psychosocial stress leads to bodily freezing, expressed by significantly less body movement compared to a stress-free control condition. For instance, we observed a lower mean velocity of the Total Body, $W = 10.0, p = 0.037, g = -0.856$ and Trunk, $W = 8.0, p = 0.021, g = -0.903$, but also of other body parts, during the TSST compared to the f-TSST (Fig. 5a). Other channels and features show similar behavior, such as a lower number of zero crossings of the Head rotation around the z-axis, i.e. in the frontal plane, $W = 6.0, p = 0.012, g = -1.174$, or a lower angular velocity energy of the Right Hand during the TSST condition, $W = 1.0, p = 0.002, g = -0.957$.

The expert features suggest that acute psychosocial stress led to static signal periods that were, on average, longer and occurred at higher percentages. Additionally, the maximum duration of static periods significantly increased during the TSST (Trunk: $W = 3.0, p = 0.003, g = 1.499$, Upper Extremities: $W = 4.0, p = 0.006, g = 1.444$, Fig. 5b).

#### Machine learning-based detection of acute psychosocial stress

When performing the classification experiments with the feature set computed over the complete (f-)TSST, the highest classification accuracy (${92.5 \pm 6.1}{\%}$) over the 5-fold model evaluation CV was achieved by the pipeline comprising *Min-Max Scaler* for feature scaling, *RFE* for feature selection, and *RF* for classification. It also achieved the highest F1-score (${92.7 \pm 6.1}{\%}$) and second-highest precision (${92.0 \pm 9.8}{\%}$) among all classification pipelines, respectively (Supplementary Information S4).

However, training the same classification pipelines with features that were extracted over the first 5min of the (f-)TSST *Interview* phase yielded considerably lower classification accuracies with the best-performing pipeline (feature scaling: *Standard Scaler*, feature selection: *RFE*, classification: *MLP*) achieving an accuracy of ${75.0 \pm 17.7}{\%}$ (F1-score: ${70.5 \pm 22.4}{\%}$, precision: ${82.0 \pm 22.2}{\%}$, Table 2).

**Table 2:** *Main study*: Mean ± standard deviation of classification performance metrics over the 5-fold model evaluation CV, trained on features extracted over the complete (f-)TSST, respectively.

Scaler	Feature selection	Classifier	Accuracy [%]	F1-score [%]	Precision [%]
Standard	SFM	RF	**71.6 (5.9)**	**71.2 (4.2)**	75.1 (13.1)
Min-Max	RFE	kNN	67.0 (8.4)	64.9 (11.2)	68.1 (8.5)
Min-Max	SkB	MLP	66.8 (6.7)	62.4 (7.5)	**75.4 (14.8)**
Min-Max	RFE	DT	65.2 (9.2)	65.9 (7.7)	66.3 (11.3)
Min-Max	SkB	NB	64.1 (10.0)	57.3 (12.2)	70.3 (13.3)
Standard	SkB	Ada	63.0 (9.3)	61.9 (7.3)	66.3 (13.2)
Min-Max	SkB	SVM	62.9 (5.6)	53.8 (11.9)	69.7 (7.0)

For each evaluated classifier, the classification pipeline combination with the highest mean accuracy is shown. The classification pipelines scoring the highest metrics are highlighted in bold.

Over the different folds, a total of $n=36$ different features ($n=5$ generic and $n=31$ expert features) were selected by the RFE algorithm. The body part groups with the most features selected were *Upper Extremities* ($n=14$ features), *Head* ($n=9$ features), and *Lower Extremities* ($n=6$ features).

The considerable drop in classification performance when only using the first 5min of the (f-)TSST *Interview* phases as information suggests that the missing *Mental Arithmetics* phase of the f-TSST induces a bias in the extracted motion features and, thus, in the classification whether an individual was exposed to the TSST of the f-TSST. As a result of these findings, we modified the f-TSST protocol in the *Main Study* to allow better comparison by adding a *Mental Arithmetics* phase with equal duration to the *Mental Arithmetics* phase in the TSST.

### Main study

#### Biopsychological response to the (f-)TSST

**Figure 6:**
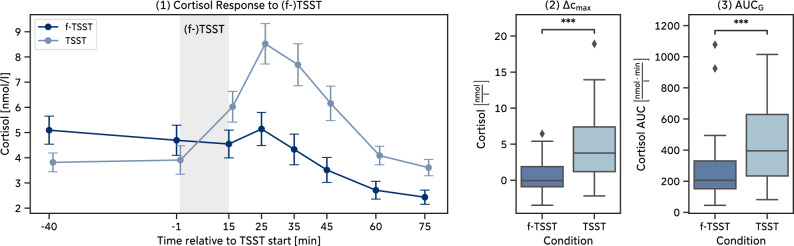
Main study: Cortisol responses to the (f-)TSST. (**1**) Mean ± standard error of cortisol samples over all participants within one condition, (**2**) maximum cortisol increase $\Delta c_{max}$, (**3**) area under the cortisol curve $\text {AUC}_{G}$.

The biopsychological response to the (f-)TSST in the *Main Study* is comparable to the response in the *Pilot Study*. Individuals who were exposed to the TSST exhibited a strong stress response, characterized by a significant cortisol increase, $W = 48.0, p < 0.001, g = 1.026$ with the peak reached 10min (*S3*) after the end of the TSST (Fig. 6-1). In contrast, the cortisol levels after the f-TSST did not increase significantly, $W = 329.0, p > 0.999, g = 0.065$, and even fell below pre-f-TSST cortisol levels after *S3*. In line with our results from the *Pilot Study*, a direct comparison between TSST and f-TSST showed that the TSST led to a significantly higher cortisol increase $\Delta c_{max}$, $W = 30.0, p < 0.001, g = 1.142$ (Fig. 6-2), and higher amounts of overall cortisol secretion, characterized by $\text {AUC}_{G}$, $W = 59.0, p < 0.001, g = 0.639$ (Fig. 6-3).

Similarly, state anxiety and negative affect both significantly *increased* after the TSST in the *Main Study* (Anxiety: $W = 57.5, p < 0.001, g = 0.826$, Negative Affect: $W = 93.0, p = 0.001, g = 0.731$) while they *decreased* after the f-TSST (Anxiety: $W = 180.5, p = 0.148, g = -0.275$, Negative Affect: $W = 101.5, p = 0.003, g = -0.502$). Directly comparing TSST and f-TSST showed that self-reported stress responses were significantly higher in the TSST compared to the f-TSST (Anxiety: $W = 84.0, p < 0.001, g = 1.174$, Negative Affect: $W = 41.5, p < 0.001, g = 1.199$).

#### Effect of acute psychosocial stress on body posture and movement

**Figure 7:**
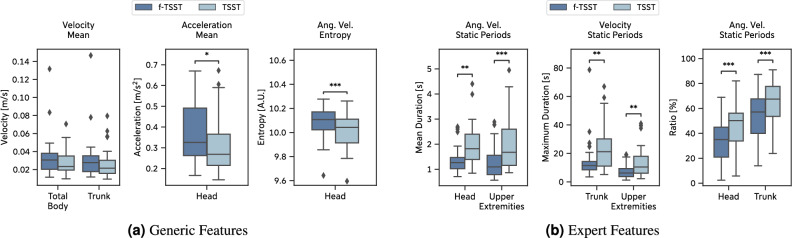
Main study: Selection of generic and expert features extracted from body posture and movements during the (f-)TSST.

The movement data from the *Main Study* show a similar behavior as the data from the *Pilot Study*. After statistical testing and Bonferroni correction, 43 (4 generic, 39 expert) out of 587 extracted features showed significant differences between TSST and f-TSST (Supplementary Information S5.1). Even though not statistically significant, the average velocity of *Total Body* and *Trunk* were lower when exposed to acute psychosocial stress. In addition, *Head* acceleration was significantly lower, $W = 107.0, p = 0.016, g = -0.478$ as well as the *Head* entropy of the angular velocity, $W = 73.0, p < 0.001, g = -0.548$ (Fig. 7a).

The expert features confirm observations from our *Pilot Study*: Periods of no movement were, on average, longer and occurred at higher percentages in the TSST compared to the f-TSST. Additionally, the duration of the longest individual static periods significantly increased during the TSST (Trunk: $W = 89.5, p = 0.004, g = 0.664$, Upper Extremities: $W = 63.0, p = 0.005, g = 0.821$, Fig. 7b). Out of the 43 statistically significant features, 16 features were extracted from the *Upper Extremities*, 10 from the *Head*, 9 from the *Total Body* and 8 from the *Trunk*.

When computing body posture and movement features over the *Interview* and *Mental Arithmetics* phases of the (f-)TSST separately, 25 out of 1158 features remaining after data cleaning were statistically significant (3 generic vs. 22 expert features, 14 *Interview* vs. 11 *Mental Arithmetics* features; Supplementary Table S5.2). The distribution across the body parts is similar compared to the features computed over the complete (f-)TSST (*Upper Extremities*: 10 features, *Head*: 7, *Total Body*: 4, *Trunk*: 4).

#### Machine learning-based detection of acute psychosocial stress

**Figure 8:**
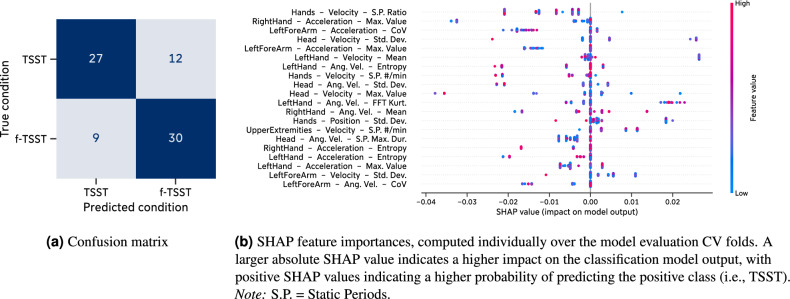
Main study: Results of best-performing classification pipeline trained on body posture and movement features computed separately over the *Interview* and *Mental Arithmetics* phases of the (f-)TSST, respectively.

The classification experiments trained on features extracted over the complete (f-)TSST of our *Main Study* data achieved a maximum classification accuracy of ${71.6 \pm 5.9}{\%}$ (F1 score: ${71.2 \pm 4.2}{\%}$, precision: ${75.1 \pm 13.1}{\%}$) by a pipeline consisting of a *Standard Scaler* for scaling, *SFM* for feature selection, and a *RF* for classification (Supplementary Information S6). We achieved a light improvement in classification performance when training the same classification pipelines on features extracted from the *Interview* and *Mental Arithmetics* phases separately, leading to a classification accuracy of ${73.4 \pm 7.7}{\%}$ (F1 score: ${71.7 \pm 9.7}{\%}$, precision: ${75.4 \pm 7.6}{\%}$, Table 3). The corresponding confusion matrix in Fig. 8a indicates that the classification of both classes (TSST and f-TSST) was equally possible, with a slightly better classification for the f-TSST condition.

For a deeper investigation into the output of our classification pipeline, we computed SHAP (SHapely Additive exPlanation) values over each model evaluation fold individual using the shap Python package (v0.44.0)^60,61^. SHAP is a game theoretic approach to explain the output of machine learning models with higher absolute SHAP values suggesting a higher impact of an individual feature on the classification model output. Positive SHAP values indicate that a feature increases the probability of the classification output being positive (in our case the TSST condition) while negative SHAP values indicate that a feature decreases the probability of the classification output being positive. Of the 40 features with the highest feature importance, 29 were generic features (11 expert features) which are distributed across different channels (acceleration: 11, angular velocity: 13, velocity: 15). The body parts contributing most to the model output were *Upper Extremities* (17 features) and *Head* (9 features), followed by *Lower Extremities* (2 features) and *Trunk* (1 feature). The 20 features with the highest SHAP values are shown in Fig. 8b.

**Table 3:** *Main study*: Mean ± standard deviation of classification performance metrics over the 5-fold model evaluation CV with features separately computed over the *Interview* and *Mental Arithmetics* phases of the (f-)TSST, respectively.

Scaler	Feature selection	Classifier	Accuracy [%]	F1-score [%]	Precision [%]
Min-Max	SFM	RF	**73.4 (7.7)**	**71.7 (9.7)**	75.4 (7.6)
Min-Max	SkB	DT	70.7 (8.8)	69.9 (12.5)	70.3 (8.8)
Min-Max	SkB	NB	68.0 (6.3)	64.2 (10.1)	71.6 (6.6)
Standard	SkB	MLP	68.0 (6.3)	63.2 (13.8)	71.2 (2.7)
Standard	SkB	kNN	67.0 (6.3)	59.1 (9.9)	**76.0 (5.6)**
Min-Max	RFE	SVM	66.8 (5.4)	64.3 (8.7)	68.3 (3.3)
Standard	RFE	Ada	63.0 (6.3)	63.5 (3.8)	64.5 (9.7)

For each evaluated classifier, the classification pipeline combination with the highest mean accuracy is shown. The classification pipelines scoring the highest metrics are highlighted in bold.

## Discussion

The main objective of our work was to explore the feasibility of detecting acute psychosocial stress from holistic body posture and movements. Along with this goal, we assessed the general influence of acute psychosocial stress on these parameters.

Since, to the best of our knowledge, our work is the first to systematically investigate the relationship between acute psychosocial stress and body posture and movements, we first had to establish a suitable experimental setup. To do so, we designed and conducted a *Pilot Study* that served as a proof-of-concept for our experimental setup. In this study, we exposed participants to the TSST and the f-TSST. Both conditions were conducted according to the original protocols^29,34^ and were performed in a randomized order. In addition, we recorded motion data using an IMU-based motion capture suit and extracted a large set of motion features to gain a picture of the behavior of individuals during the (f-)TSST that is as comprehensive as possible.

The results of our *Pilot Study* suggest that we successfully induced acute psychosocial stress using the TSST protocol. The TSST provoked a multidimensional stress response leading to strong HPA axis activation as well as increases in perceived negative affect and state anxiety, which is in line with existing literature^10,37,62^. In contrast, the f-TSST served as a reliable, stress-free control condition in a comparable scenario without activating the HPA axis and increases in negative affect and anxiety. This also reproduces previous studies conducting the f-TSST^34,45,46^. In addition to the established markers for assessing the biopsychological stress response, the body posture and movement features extracted during the (f-)TSST showed significant differences between the TSST and f-TSST. This indicates that acute psychosocial stress affects body posture and movements. Overall, individuals showed slower movements during the TSST, indicated by a significantly decreased mean velocity and angular velocity in most body parts. Additionally, fewer movements occurred during the TSST with longer breaks in between movement phases.

Our initial classification experiments achieved a high accuracy (${92.5 \pm 6.1}{\%}$ for the best-performing pipeline). However, a more detailed investigation suggested that the high classification performance could be confounded by the bias in the protocols of the TSST and f-TSST. While the TSST consists of a *Interview* and a *Mental Arithmetic* phase, each lasting 5min, the f-TSST only consists of an *Interview* phase lasting 10min. We mitigated this bias by only using the first 5min of the *Interview* phase of the TSST and f-TSST for the classification, resulting in an accuracy of ${75.0 \pm 17.7}{\%}$ for the best-performing pipeline using unbiased features from comparable phases of the study protocol. The results of our classification experiments suggest a strong link between body posture and movement information and indicate that they can potentially be used for acute stress detection. Thus, it motivated us to further investigate the relationship between acute psychosocial stress and body posture and movements in our *Main Study*.

Apart from the imbalanced study protocol, our *Pilot Study* suffered from additional limitations. First of all, the relatively small number of individuals included in the final analysis ($N=20$) and the imbalanced gender distribution (90% female) limits the generalizability of our findings. In addition, we did not control for the use of hormonal contraceptives and the menstrual cycle phase among female participants, which both have been shown to influence the cortisol response to acute stress^40^. Lastly, the *Perception Neuron* IMU-based motion capture suit was suffering from positional and rotational drifts and the quality of the recorded motion capture data heavily depended on the quality of the performed calibration, especially during the *Steady pose*. Even though the experimenters paid special attention to the calibration poses being performed accurately, potential effects on the resulting data can not be fully ruled out. The drifts varied across participants and partly required manual post-processing of the recorded motion data. This procedure could have affected the quality of the recorded motion data and might have led to the loss of some relevant motion information. Further, the sensor nodes of the system were interconnected via cables, which might have encumbered participants’ movements. Despite the aforementioned limitations, we expect the main findings of our *Pilot Study* not to be compromised. Firstly, the average cortisol response was comparable to those of previous TSST studies^37^, indicating successful HPA axis activation. Secondly, the changes in body posture and movements were consistent throughout participants, regardless of the amount of drift. Nonetheless, we addressed these limitations in our second study to improve the methodological robustness and enhance the reliability of the data.

After synthesizing the findings of our *Pilot Study*, we designed our *Main Study*. The main changes in this study were a more thorough recruiting and screening procedure (as outlined in Sect. Data acquisition) to ensure a more balanced sample and the use of a modified study protocol. We shortened the *Interview* phase of the f-TSST to 5min and added a *Mental Arithmetics* phase from the Placebo TSST^48^ in which participants should count upwards from 0 in steps of 15 to avoid bias in the extracted features due to the unbalanced study protocol. In addition, we used the *Xsens* motion capture system as an improved sensor-based motion capture solution that did not exhibit any drifts and was less obtrusive since the sensors were attached to the body using elastic straps.

The biopsychological response to the (f-)TSST in our *Main Study* was comparable to that of our *Pilot Study*. The TSST induced a strong HPA axis activation as well as increases in negative affect and state anxiety. In contrast, even though the additional *Mental Arithmetics* phase could have been a potentially stressful situation for some individuals, it did not activate the HPA axis and did lead to increased negative affect or anxiety. In addition to the established markers, the body posture and movement features extracted during the (f-)TSST reproduce the findings of our *Pilot Study*.

When looking at the results of the statistical analyses, it becomes apparent that features characterizing movements by *Upper Extremities* (hands, forearms, and arms) and the *Head* showed the largest effect sizes, indicating that they were affected most by the acute psychosocial stress situation. Hand movements during the TSST were characterized by a longer mean and maximum duration of static periods. Additionally, the percentage of time without movement was higher, which resulted in decreased hand movement compared to the f-TSST. In contrast, we observed considerably more hand gesturing during the f-TSST. Similar behavior was also observed by Pisanski et al., who have shown that exposure to acute psychosocial stress leads to a reduction in arm movements compared to a pre-stress baseline^28^. This is further supported when consulting the videos acquired during the (f-)TSST, in which we observed that some participants during the TSST either put their hands folded in front of their bodies or kept their arms straight next to their bodies.

Apart from hand movements, exposure to acute psychosocial stress led to less trunk and total body movement as well as fewer movement variations. A reduction in trunk movement is related to decreased body sway, which, together with decreased heart rate, is an indicator of bodily freezing that occurs in situations of social threat, such as generated by the TSST^18,25,26^. Furthermore, comparing the behavior between the TSST and f-TSST using the acquired video recordings revealed that individuals during the TSST were more likely to drop their heads, which is an indicator of negative emotions^22^ that are induced by the TSST^63^. Additionally, participants in the TSST repeatedly attempted to avoid eye contact with the evaluation panel by directing their gaze to the corners of the testing room, even though they were repeatedly reminded to keep eye contact. This behavior is also reflected by the extracted head movement features, which differed significantly between the TSST and the f-TSST.

Summarized, the findings of our studies confirm several previous studies that investigated the relationship between acute stress and single aspects of body posture and movements. Thus, our results suggest a close interplay between behavioral, endocrinological, and motoric systems during situations of acute stress and motivate the use of motion information as an additional digital biomarker to the established biopsychological markers for acute stress assessment to obtain a more holistic picture of the human stress response.

The sole use of motion information to detect acute psychosocial stress using machine learning (partly) supports this conjecture. We achieved a maximum accuracy of ${73.4 \pm 7.7}{\%}$ for detecting whether an individual was exposed to acute stress or not. This classification result is comparable to results from previous studies. For example, Arnrich et al.^21^ differentiated acute stress from cognitive load from pressure distributions from a chair with an accuracy of 73.8% while van der Zee et al. achieved an accuracy of 74.4% for detecting whether individuals were lying or telling the truth^20^. In contrast to both approaches, we used nested cross-validation for hyperparameter tuning and model evaluation to obtain a less biased estimate of the generalization performance of our classification pipelines^55^. The relatively low standard deviation between the accuracy of the model evaluation CV folds supports the generalizability of our results.

A deeper investigation into the feature importances indicated that the body parts with the highest contributions to the classification output are the *Head* and the *Upper Extremities*, which is in line with the results of our *Pilot Study*, the statistical analyses of our *Main Study*, the observations of the panel members and assessment of video recordings, and the findings from studies such as Vrij et al.^19^ and Pisanski et al.^28^. This suggests that the machine learning model learned to distinguish between the TSST and f-TSST by learning the differences in the behavior of individuals during the two conditions. Even though the same trends are visible in the generic and expert-based feature sets, our statistical analyses revealed that more expert features characterizing body posture and movements showed statistically significant differences between the (f-)TSST compared to the generic features. In addition, the expert features showed larger effect sizes. This highlights the complex behavioral changes induced by acute psychosocial stress and the resulting necessity of domain knowledge to understand how stress affects body posture and movements and how these changes can be characterized. Furthermore, it motivates exploring additional expert features that are not included in our current feature set. An alternative approach to this challenge could be the use of deep learning-based approaches that can learn the relevant features from the data itself. This could potentially lead to a more robust and generalizable classification model that might be able to learn more complex patterns in the data not visible to the human eye. In this initial explorative work, we chose to rely on “traditional” machine learning approaches due to the rather limited sample size and the easier interpretation of the model outputs.

In our experiments, the classification performance was higher when using features extracted over the *Interview* and *Mental Arithmetics* phases of the (f-)TSST separately compared to features extracted over the entire (f-)TSST. This suggests that the social-evaluative threat induced during the TSST affects body posture and movements in a different way during the two phases. This is also supported by the statistical analysis results: While the number of significant features from the *Interview* and *Mental Arithmetics* phases were roughly equal, it was not the same set of features that were significant in *both* phases (Supplementary Table S5.2). This is an important finding for future work that should further investigate the effect of acute psychosocial stress on body posture and movements in different scenarios.

However, the classification results also indicate that the sole use of motion information can, so far, not be used to reliably detect acute psychosocial stress. One possible explanation for this is that both acute psychosocial stress and human movement are complex and multifaceted phenomena that are affected by various factors. While we saw a reduction in movement in almost every study participant, the general amount of movement is highly individual, making it difficult to detect stress from body posture and movements without having a motion baseline measurement that determines the general movement behavior of an individual. Further indication for this high inter-subject variability is given by the SHAP feature importance analysis which shows that *Head* and *Upper Extremities* are the most important body parts for classification. However, the individual contributions of the single features vary considerably between individuals.

In contrast to our approach, the majority of previous studies investigating stress-induced changes lack proper control conditions that are stress-free on the one hand but are as similar as possible to the stressor on the other hand^28,64,65^. For instance, Pisanski et al. investigated stress-induced changes in digital biomarkers (such as voice, facial expressions, or movements) by comparing the TSST to a *pre-stress* baseline^28^. This can potentially induce a classification bias since the tasks performed during the TSST and the baseline measurement are different. Thus, parts of the observed differences in the digital biomarkers could be attributed to the different tasks, or to sequence effects, and not to the stress situation. The findings of our *Pilot Study*, showing the considerable influence of the current task on the movement patterns of individuals, as well as the discrepancies in significant motion features between the two (f-)TSST phases of our *Main Study* might support this argument. For that reason, we selected the f-TSST and modified it in a way that it is as similar as possible to the TSST. This allows us to precisely study the effect of social-evaluative threat on body posture and movement since the presence of social-evaluative threat was the main difference between the f-TSST and the TSST without any biases due to different tasks.

In our study, the use of IMU-based motion capture systems appeared to be an optimal trade-off between accurate measurement of movement and unobtrusiveness. However, wearing such a suit still bears the possibility to affect the movement behavior of individuals. While the *Xsens* suit used in our *Main Study* already poses a considerable improvement over the *Perception Neuron* suit used in our *Pilot Study*, it still consists of several sensors attached to the body via straps which might affect participants’ movements, especially on the first study day. After the study, participants reported that they did not feel very restricted in their range of motion during the study and that they got used to the suit quite quickly. However, it is still possible that the suit could slightly affect the movement behavior of individuals. Since individuals wore the same suit in both the TSST and f-TSST, we expect that a possible effect of the suit on the movement behavior of individuals was similar in both conditions.

Nonetheless, the condition order might still influence the movement behavior of individuals due to the novelty of the scenario on the first study day, regardless of the condition (TSST or f-TSST). We chose not to include the condition order as a feature in our classification experiments since we did not want the classification model to learn the condition order as a confounding factor. When comparing the prediction probability of the classification model between the condition orders, we observed a slightly higher prediction confidence for the condition that was performed on the *first* day, which could be attributed to the novelty and unpredictability effect mentioned above, leading to a similar movement reduction as acute stress does. Even though the effect was small, future work should investigate the effect of the condition order on the movement behavior of individuals in more detail.

To avoid potential movement changes due to our chosen motion capture approach, we will explore other, less obtrusive motion capture modalities in future work, such as radar- or video-based methods. Radar-based approaches have the advantage of not only being able to capture macroscopic movements, such as body posture^66,67^ but also microscopic movements caused by heart sounds or respiration^15,68^. On the other hand, video-based approaches, such as AlphaPose^69^ or OpenPose^70^ could be employed for unobtrusive motion capturing during the (f-)TSST. While they might also come with the drawback of being less accurate than established motion capture techniques, particularly the video-based approach is worth investigating in more detail as soon as motion features have become an established behavioral marker for stress assessment since it is low-cost and easy to employ. In addition, the TSST protocol mandates video recordings to be taken during the examination. Thus, extracting body posture and movements from video-based motion capturing could enable widespread use in many biopsychological studies without the need to purchase expensive equipment.

Generally, the ambiguous definition of “stress” remains a major challenge in stress research^71^. In our work, we focused on psychosocial stress because it is one of the most common stressors in everyday life^3,71^. Further, acute psychosocial stress causes the strongest HPA axis activation^24^. Understanding the determinants of HPA axis activity is crucial since it is the most promising biological pathway to understand the transition from stress to disease^72^. This differentiates our work from other approaches that presented solutions for “stress detection” using unobtrusive modalities, such as smartphone data^73,74^, keyboard typing or mouse movements^75,76^, or radio frequency waves, such as WiFi or millimeter radar^15,77,78^. Even though these studies claimed to induce stress and detect these stressful situations reliably, none of them assessed the HPA axis activity by measuring the cortisol response to stress. Instead, they mostly relied on subjective stress ratings to determine whether individuals were stressed or not. Furthermore, the stress induction was mostly restricted to protocols that induce cognitive load instead of acute stress. While all of these approaches are valid and present valuable contributions to stress research, it makes it difficult to compare them with our presented study since both situations of cognitive load and acute psychosocial stress can trigger physiological arousal.

In our case, the f-TSST would also induce physiological arousal since it has been shown to activate the SNS, leading to changes in heart rate (variability) and alpha-amylase^34^. However, since it does not activate the HPA axis, we considered the f-TSST not to be *stressful* according to our definition. To better understand and investigate the negative effects of stress on health, it is crucial to properly distinguish these two situations from each other^79^. We are aware that the definition of “stress” is still ambiguous and that there is no consensus on a single definition that satisfies all research fields^71^. In our work, we used the condition (TSST vs. f-TSST) as the ground truth label to define whether an individual was stressed or not. This is a simplification of the actual problem since “stress” is not a binary condition but rather a continuous variable. Even though the TSST is considered the gold standard for inducing acute psychosocial stress^24^, leading to the strongest HPA axis activation, it has been shown that not every individual shows a cortisol response to the TSST^80^. Not only does this mean that people can be stressed without showing a cortisol response it also indicates that a classification into “stressed” and “not stressed” based on the cortisol response, the supposed gold standard neuroendocrine marker for an acute stress response, can also lead to a considerable number of misclassifications. In future work, we plan to address this ambiguity systematically by exploring the effect of different stress definitions on our results, similar to the approach of Norden et al.^65^. In addition, we plan to directly investigate the relationship between individual stress-induced movement alterations and the cortisol responses to the (f-)TSST. Furthermore, we plan to extend our research to different scenarios that are not only restricted to the laboratory TSST protocol. For instance, it would be interesting to investigate changes in full-body posture and movements during daily-life scenarios, such as workplace stressors, which could be an extension to the work of Arnrich et al.^21^.

We are convinced that our findings demonstrate the potential of using body posture and movement information to assess acute stress reactions since it might help to better distinguish phases of cognitive load from acute stress situations that include social-evaluative stress. Even though extracting meaningful information characterizing the behavior of individuals during acute stress situations is a challenging task, we believe that the presented work is a first step towards a more holistic picture of the human stress response. Establishing these markers as a complement to the well-known biopsychological makers can deliver additional insights into the bodily response to acute psychosocial stress. Since these markers can be measured more easily in the wild compared to traditional biomarkers, including motion information when assessing the human stress response might help to better understand the link between behavioral, physiological, and motoric processes during stress.

## Conclusion and outlook

In our work, we assessed the influence of acute psychosocial stress on body posture and movements by exposing individuals to acute stress (TSST) and a stress-free control condition (f-TSST) while measuring full-body motion. Our findings show that situations of social-evaluative threat and uncontrollability, as administered by the TSST, lead to behavioral changes compared to the f-TSST, which were reproducible over two studies. These changes are characterized by slower movements and by periods of no movement that are, on average, longer and occur more often. In particular, we observed this movement reduction for head, upper extremities, and upper body movements. Using movement features extracted separately over the *Interview* and *Mental Arithmetics* phases of the (f-)TSST, we were able to detect whether an individual was exposed to acute stress or not with an accuracy of ${73.4 \pm 7.7}{\%}$.

Our results show the feasibility of using movement information to detect phases of acute stress solely from movement information. To the best of our knowledge, we are the first to systematically investigate how acute psychosocial stress affects body posture and movements. While future work is necessary to validate our findings further we are convinced that our results are an important step to providing valuable insights into the underlying biopsychological mechanisms. Future research should continue to examine the connection between body movements and inner processes, such as examining the link between movements and inflammation as a response to acute psychosocial stress. Complementary, other movement-based indicators of human emotions, such as facial expressions, can be explored.

Ultimately, the study of the relationship between acute psychosocial stress and body posture and movements can deepen our understanding of the human stress response and its effects on the body and mind. Thus, they can contribute to establishing body movements as a novel digital biomarker for assessing the human stress response more holistically. This can lead to the development of practical applications, such as interventions to mitigate the negative effects of stress on physical and mental health.

## Supplementary Information

Supplementary Information.

## Data Availability

All (raw) data recorded during this study are available on OSF (*Pilot Study*: https://osf.io/qvzdg/ and *Main Study* https://osf.io/va6t3/. The source code for data processing and for reproducing all analysis results and figures is available on *GitHub*: https://github.com/empkins/stresspose-analysis.
