# Supplementary material for: Machine learning-based detection of acute psychosocial stress from body posture and movements
Source: Sci Rep. 2024 Apr 8;14:8251. doi: 10.1038/s41598-024-59043-1 (PMC11375162; doi:10.1038/s41598-024-59043-1)
Supplement: Supplementary file 1 — Supplementary Information. [file 41598_2024_59043_MOESM1_ESM.pdf]

Supplementary Information for Paper "StressPose - Detection of Acute Psychosocial Stress from Body Posture and Movements"

R. Richer, V. Koch, L. Abel, F. Hauck, M. Kurz, V. Ringgold, V. Müller,
A. Küderle, L. Schindler-Gmelch, N. Rohleder, B. M. Eskofier

1 Additional Information

Perception Neuron Preprocessing Pipeline

As the motion capture data were suffering from sensor drifts in position and rotation, we first preprocessed the data before further processing. We removed position drifts by filtering the `pos` channel with a third-order Butterworth high-pass filter and a cutoff frequency of $f_c = 0.01\,\text{Hz}$. Due to the hierarchical structure of the motion capture data format, we only filtered data from the root node (*Hips*) as the positions of all other body parts were defined relative to the root node. As a similar approach was not possible to filter the rotation drifts of Euler angles, we developed the following rotation drift filtering pipeline (Figure 1):

1. Unwrap the rotation values given as radian phases $\varphi, \varphi \in [-\pi, +\pi]$ by adding multiples of $\pm 2\pi$ such that adjacent angle differences $\Delta\varphi$ are never greater than π.
2. Convert rotation data from Euler angles to quaternions.
3. Approximate the rotation drift by applying high-pass Butterworth filters with cutoff frequencies f_c in the range 0.01–0.06 Hz and subtracting the filtered data from the original data.
4. Remove drift by rotating the original data by the approximated drift.
5. Convert quaternions back to Euler angles.

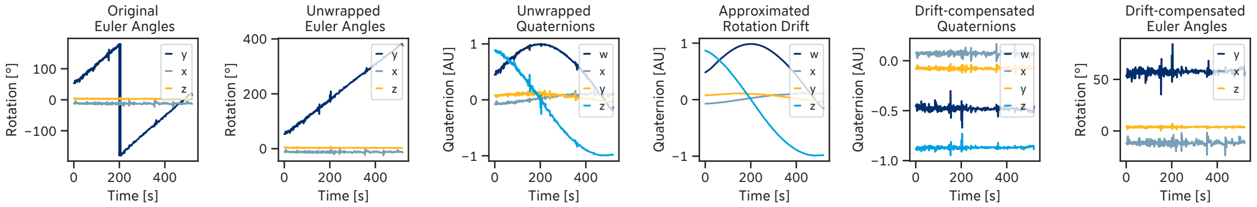

Filtering pipeline to remove rotation drifts from the motion capture data.

Since the rotation drifts were different for each recording regarding the magnitude and the affected body parts, we empirically determined and applied custom filter parameters for each recording. A list of the individual filter parameters can be found in the dataset description [1, 2, 3]. Afterwards, we computed the frame-wise global position `pos_global` of each body part from the drift-corrected data according to

$$x'^j = \mathbf{M}^j_{global} \cdot x^j, \tag{1}$$

where $x^j, x \in \mathbb{R}^4$ and $x'^j, x' \in \mathbb{R}^4$ describe the *local* and *global* position for one body part j in homogeneous coordinates, respectively, and $\mathbf{M}^j_{global}, \mathbf{M} \in \mathbb{R}^{4x4}$ is the transformation matrix consisting of rotation R and translation T (Equation 2).

$$\mathbf{M}_{global} = \begin{bmatrix} R_{11} & R_{12} & R_{13} & T_x \\ R_{21} & R_{22} & R_{23} & T_y \\ R_{31} & R_{32} & R_{33} & T_z \\ 0 & 0 & 0 & 1 \end{bmatrix} \tag{2}$$

Due to the hierarchical structure for the motion capture data format, the global transformation matrix $\mathbf{M}^j_{global}$ for a given body part j is obtained by recursively multiplying the local transformation matrix $\mathbf{M}^j_{local}$

with the global transformation matrix of the parent $\mathbf{M}^{j-1}_{global}$ as depicted in Equation 3, where $i = 0$ indicates the root node of the hierarchy (*Hips*).

$$\mathbf{M}^{j}_{global} = \prod_{i=0}^{j} \mathbf{M}^{i}_{local} \tag{3}$$

Additionally, we extracted the global rotation `rot_glo` as Euler angles from the rotation information R in the global transformation matrix $\mathbf{M}_{global}$.

2 Additional Tables

Overview on Extracted Generic Features

Supplementary Table S1.1: Pilot Study

Pilot Study: Overview of generic features computed per body part (group) from the *Perception Neuron* system.

Body Part (Group)	Channels			Axes		Generic Features
	`vel`	`ang_vel`	`rot`	x, y, z	L2-norm	
Head	✓	✓	✓	✓	✓	*all*
Chest	✓	✓	✓	✓	✓	*all*
{Left/Right} Hand	✓	✓	✓	✓	✓	*all*
{Left/Right} ForeArm	✓	✓	✓	✗	✓	`mean`, `std`, `cov`, `max_val`
{Left/Right} Leg	✓	✓	✓	✗	✓	`mean`, `std`, `cov`, `max_val`
Trunk	✓	✓	✗	✗	✓	*all*, except `z_cross` and `m_cross`
Upper Extremities	✓	✓	✗	✗	✓	*all*, except `z_cross` and `m_cross`
Lower Extremities	✓	✓	✗	✗	✓	*all*, except `z_cross` and `m_cross`
Total Body	✓	✓	✗	✗	✓	*all*, except `z_cross` and `m_cross`

Supplementary Table S1.2: Main Study

Main Study: Overview of generic features computed per body part (group) from the *Xsens* system.

Body Part (Group)	Channels				Axes		Generic Features
	`acc`	`vel`	`ang_vel`	`rot`	x, y, z	L2-norm	
Head	✓	✓	✓	✓	✓	✓	*all*
Chest (T8)	✓	✓	✓	✗	✓	✓	*all*
{Left/Right} Hand	✓	✓	✓	✓	✓	✓	*all*
{Left/Right} ForeArm	✓	✓	✓	✓	✗	✓	`mean`, `std`, `cov`, `max_val`
{Left/Right} Leg	✓	✓	✓	✓	✗	✓	`mean`, `std`, `cov`, `max_val`
Trunk	✓	✓	✓	✗	✗	✓	*all*, except `z_cross` and `m_cross`
Upper Extremities	✓	✓	✓	✗	✗	✓	*all*, except `z_cross` and `m_cross`
Lower Extremities	✓	✓	✓	✗	✗	✓	*all*, except `z_cross` and `m_cross`
Total Body	✓	✓	✓	✗	✗	✓	*all*, except `z_cross` and `m_cross`

Overview on Extracted Expert Features

Supplementary Table S1.3: Pilot Study

Pilot Study: Overview of expert features computed per body part (group) from the *Perception Neuron* system.

Body Part (Group)	Channels			Expert Features
	`rot_glo`	`vel`	`ang_vel`	
Chest	✗	✓	✓	`Static Periods`
Left Hand & Right Hand	✗	✓	✓	
Head	✓	✓	✓	`Absolute Movement`, `Static Periods`
Trunk	✓	✓	✓	
Upper Extremities	✓	✓	✓	
Lower Extremities	✓	✓	✓	

Supplementary Table S1.4: Main Study

Main Study: Overview of expert features computed per body part (group) from the *Xsens* system.

Body Part (Group)	Channels		Expert Features
	`vel`	`ang_vel`	
Chest (T8)	✓	✓	`Static Periods`
Left Hand & Right Hand	✓	✓	
Head	✓	✓	`Absolute Movement`, `Static Periods`
Trunk	✓	✓	
Upper Extremities	✓	✓	
Lower Extremities	✓	✓	

Supplementary Table S2: Hyperparameter Search Space

Grid-search space for the individual feature selection and classification algorithms of the *StressPose* pipeline.

Type	Algorithm	Parameter	Values
Feature Selection	SkB	`k`	$\{2, 4, ..., 18\}$
	RFE	`n_features`	$\{2, 4, ..., 18\}$
Classifier	NB	–	–
	kNN	`n_neighbors`	$\{1, 3, ..., 19\}$
		`weights`	[“uniform”, “distance”]
	DT	`criterion`	[“gini”, “entropy”]
		`max_depth`	$\{1, 3, ..., 19\}$
		`min_samples_leaf`	$\{0.1, 0.2, 0.3, 0.4\}$
		`min_samples_split`	$\{0.1, 0.2, ..., 0.7\}$
		`max_features`	$\{0.1, 0.2, 0.3, 0.4, 0.5,$ “auto”, “log2”, None$\}$
	SVM-lin	`C`	$\{10^{-2}, 10^{-1}, ..., 10^{4}\}$
	SVM-rbf	`C`	$\{10^{-2}, 10^{-1}, ..., 10^{4}\}$
		`gamma`	$\{10^{-4}, 10^{-3}, ..., 10^{3}\}$
	SVM-poly	`C`	$\{10^{-2}, 10^{-1}, ..., 10^{4}\}$
		`degree`	$\{2, 3, 4, 5\}$
	MLP	`hidden_layer_sizes`	$[(1), (1, 1), (1, 1, 1), (2), (2, 2), (2, 2, 2), (5), (5, 5), (5, 5, 5)]$
		`activation`	[“identitiy”, “tanh”, “relu”]
		`solver`	[“lbfgs”, “adam”]
		`alpha`	$\{10^{-2}, 10^{-1}, ..., 10^{2}\}$
	Ada	`base_estimator`	[DT, SVC]
		`n_estimators`	$\{10, 20, ..., 500\}$
		`learning_rate`	$\{0.01, 0.02, ..., 0.1, 0.2, ..., 1.5\}$
	RF	`criterion`	[“entropy”]
		`max_depth`	$\{4, 8, 12, ..., 48,$ None$\}$
		`max_features`	$\{0.1, 0.2, 0.3, 0.4,$ “sqrt”$\}$
		`min_samples_leaf`	$\{0.05, 0.1, 0.15, 0.20\}$
		`min_samples_split`	$\{0.1, 0.2, 0.3, 0.4\}$
		`min_weight_fraction_leaf`	$\{0.1, 0.2, 0.3, 0.4\}$
		`max_leaf_nodes`	$\{2, 4, ..., 18\}$
		`min_impurity_decrease`	$\{0, 0.01, ..., 0.09\}$
		`n_estimators`	$\{10, 20, ..., 390\}$
		`ccp_alpha`	$\{0, 0.01, ..., 0.09\}$

Supplementary Table S3: Statistical Test Results – *Pilot Study*

Features over entire (f-)TSST

Results of statistical tests of extracted body posture and movement features between TSST and f-TSST from the *Pilot Study*. p-values are corrected for multiple comparisons using the Bonferroni method. *Note*: Only motion features with statistically significant ($p < 0.05$) differences are shown.

Feature Type	*Body Part*	*Channel*	*Type*	*Metric*	W	p	Hedges' g
Expert	*Lower Extremities*	*Ang. Vel.*	*Static Periods*	*Std. Duration (s)*	0	<0.001***	0.746
				Max. Duration (s)	0	<0.001***	0.741
				Mean Duration (s)	0	<0.001***	0.934
Generic	*Right Fore Arm*	*Ang. Vel.*	*CoV*	*CoV*	0	<0.001***	1.421
	Right Hand	*Ang. Vel.*	*CoV*	*CoV*	0	<0.001***	1.421
Expert	*Head*	*Velocity*	*Static Periods*	*Std. Duration (s)*	0	<0.001***	1.159
	Left Hand & Right Hand	*Velocity*	*Static Periods*	*Std. Duration (s)*	0	<0.001***	1.122
	Head	*Velocity*	*Static Periods*	*Mean Duration (s)*	0	<0.001***	1.065
	Left Hand & Right Hand	*Velocity*	*Static Periods*	*Mean Duration (s)*	0	<0.001***	1.020
	Lower Extremities	*Velocity*	*Static Periods*	*Std. Duration (s)*	0	<0.001***	0.843
	Head	*Ang. Vel.*	*Static Periods*	*Std. Duration (s)*	0	<0.001***	1.066
				Mean Duration (s)	0	<0.001***	1.524
				Max. Duration (s)	0	<0.001***	0.904
	Lower Extremities	*Velocity*	*Static Periods*	*Mean Duration (s)*	0	<0.001***	0.659
Generic	*Right Fore Arm*	*Ang. Vel.*	*Mean*	*Mean*	1	0.002**	−1.549
Expert	*Chest*	*Ang. Vel.*	*Static Periods*	*Mean Duration (s)*	1	0.002**	1.496
Generic	*Right Fore Arm*	*Velocity*	*Mean*	*Mean*	1	0.002**	−1.085
	Right Hand	*Ang. Vel.*	*Mean*	*Mean*	1	0.002**	−1.549
Expert	*Lower Extremities*	*Velocity*	*Static Periods*	*Max. Duration (s)*	1	0.002**	1.064
	Left Hand & Right Hand	*Velocity*	*Static Periods*	*Max. Duration (s)*	1	0.002**	1.089
	Upper Extremities	*Velocity*	*Static Periods*	*Ratio (%)*	1	0.002**	1.321
Generic	*Right Hand*	*Ang. Vel.*	*Abs. Energy*	*Abs. Energy*	1	0.002**	−0.957
Expert	*Head*	*Ang. Vel.*	*Static Periods*	*Ratio (%)*	1	0.002**	1.478
	Trunk	*Ang. Vel.*	*Static Periods*	*Mean Duration (s)*	2	0.002**	1.283
	Chest	*Ang. Vel.*	*Static Periods*	*Std. Duration (s)*	3	0.004**	0.963
	Trunk	*Ang. Vel.*	*Static Periods*	*Max. Duration (s)*	3	0.004**	1.499
				Ratio (%)	3	0.004**	1.375
	Lower Extremities	*Ang. Vel.*	*Static Periods*	*Ratio (%)*	3	0.004**	0.978
	Left Hand & Right Hand	*Ang. Vel.*	*Static Periods*	*Mean Duration (s)*	3	0.004**	0.888
	Trunk	*Ang. Vel.*	*Static Periods*	*Std. Duration (s)*	3	0.004**	1.181
	Head	*Velocity*	*Static Periods*	*Max. Duration (s)*	3	0.004**	1.168
	Trunk	*Velocity*	*Static Periods*	*Max. Duration (s)*	4	0.006**	1.097
	Left Hand & Right Hand	*Velocity*	*Static Periods*	*Ratio (%)*	4	0.006**	1.242
	Upper Extremities	*Velocity*	*Static Periods*	*Max. Duration (s)*	4	0.006**	1.444
	Lower Extremities	*Velocity*	*Static Periods*	*Ratio (%)*	5	0.008**	0.980
	Chest	*Ang. Vel.*	*Static Periods*	*Max. Duration (s)*	5	0.008**	1.120
	Head	*Velocity*	*Static Periods*	*Ratio (%)*	5	0.008**	1.230
	Upper Extremities	*Velocity*	*Static Periods*	*Mean Duration (s)*	6	0.011*	1.622
	Trunk	*Velocity*	*Static Periods*	*Std. Duration (s)*	6	0.011*	1.376
	Left Hand & Right Hand	*Ang. Vel.*	*Static Periods*	*Std. Duration (s)*	6	0.011*	0.581
				Max. Duration (s)	6	0.011*	0.772
	Chest	*Velocity*	*Static Periods*	*Max. Duration (s)*	7	0.015*	1.137
	Upper Extremities	*Ang. Vel.*	*Static Periods*	*Ratio (%)*	7	0.015*	1.433
	Left Hand & Right Hand	*Ang. Vel.*	*Static Periods*	*Ratio (%)*	7	0.015*	1.484
Generic	*Left Fore Arm*	*Rotation*	*Max. Value*	*Max. Value*	7	0.015*	−1.012
Expert	*Chest*	*Ang. Vel.*	*Static Periods*	*Ratio (%)*	7	0.015*	1.296
		Velocity	*Static Periods*	*Std. Duration (s)*	8	0.020*	1.341
Generic	*Trunk*	*Velocity*	*Mean*	*Mean*	8	0.020*	−0.903
Expert	*Trunk*	*Velocity*	*Static Periods*	*Mean Duration (s)*	8	0.020*	1.462
	Upper Extremities	*Velocity*	*Static Periods*	*Std. Duration (s)*	9	0.027*	1.528
Generic	*Total Body*	*Ang. Vel.*	*CoV*	*CoV*	9	0.027*	0.869
Expert	*Upper Extremities*	*Ang. Vel.*	*Static Periods*	*Counts per Minute*	9	0.027*	1.281
Generic	*Left Fore Arm*	*Rotation*	*Std. Dev.*	*Std. Dev.*	10	0.035*	−1.083
	Total Body	*Velocity*	*Mean*	*Mean*	10	0.035*	−0.856
	Head	*Rotation*	*Zero Crossings*	*Zero Crossings*	10	0.035*	−1.063
	Trunk	*Ang. Vel.*	*CoV*	*CoV*	11	0.045*	0.901
	Total Body	*Ang. Vel.*	*FFT Aggregated Skewness*	*FFT Skewness*	11	0.045*	−0.968

Supplementary Table S4: Classification Results – *Pilot Study*

Features over entire (f-)TSST

Mean ± standard deviation of classification performance metrics over the 5-fold model evaluation CV with features computed over the complete (f-)TSST procedure. For each evaluated classifier, the classification pipeline combination with the highest mean accuracy is shown. The classification pipelines scoring the highest metrics are highlighted in **bold**.

Scaler	Feature Selection	Classifier	Accuracy [%]	F1-score [%]	Precision [%]
Min-Max	RFE	RF	**92.5 ± 6.1**	**92.7 ± 6.1**	92.0 ± 9.8
Standard	RFE	kNN	87.5 ± 0.0	86.3 ± 1.3	**96.0 ± 8.0**
Standard	SkB	MLP	85.0 ± 12.2	85.4 ± 10.7	88.0 ± 16.0
Min-Max	SkB	Ada	82.5 ± 6.1	80.4 ± 8.3	91.0 ± 11.1
Standard	SkB	SVM	82.5 ± 10.0	83.8 ± 8.7	80.0 ± 12.6
Standard	SkB	NB	80.0 ± 10.0	78.7 ± 9.9	88.0 ± 16.0
Min-Max	RFE	DT	77.5 ± 9.4	73.9 ± 17.6	84.3 ± 13.5

Statistical Test Results – *Main Study*

Supplementary Table S5.1: Features over entire (f-)TSST

Results of statistical tests of extracted body posture and movement features between TSST and f-TSST from the *Main Study*. p-values are corrected for multiple comparisons using the Bonferroni method. *Note*: Only motion features with statistically significant ($p < 0.05$) differences are shown.

Feature Type	*Body Part*	*Channel*	*Type*	*Metric*	W	p	Hedges' g
Expert	*Upper Extremities*	*Ang. Vel.*	*Static Periods*	*Mean Duration (s)*	17	<0.001***	0.823
	Left Hand & Right Hand	*Ang. Vel.*	*Static Periods*	*Mean Duration (s)*	17	<0.001***	0.825
	Total Body	*Ang. Vel.*	*Static Periods*	*Ratio (%)*	34	<0.001***	0.711
Generic	*Head*	*Ang. Vel.*	*CoV*	*CoV*	35	<0.001***	0.611
Expert	*Upper Extremities*	*Ang. Vel.*	*Static Periods*	*Ratio (%)*	39	<0.001***	0.618
	Total Body	*Ang. Vel.*	*Static Periods*	*Mean Duration (s)*	39	<0.001***	0.906
	Left Hand & Right Hand	*Ang. Vel.*	*Static Periods*	*Ratio (%)*	47	<0.001***	0.581
	Total Body	*Ang. Vel.*	*Static Periods*	*Std. Duration (s)*	50	<0.001***	0.858
	Head	*Ang. Vel.*	*Static Periods*	*Ratio (%)*	56	<0.001***	0.672
	Total Body	*Ang. Vel.*	*Static Periods*	*Max. Duration (s)*	58	<0.001***	0.799
	Left Hand & Right Hand	*Ang. Vel.*	*Static Periods*	*Std. Duration (s)*	62	<0.001***	0.598
	Upper Extremities	*Ang. Vel.*	*Static Periods*	*Std. Duration (s)*	63	<0.001***	0.757
	Trunk	*Ang. Vel.*	*Static Periods*	*Std. Duration (s)*	68	<0.001***	0.696
				Mean Duration (s)	70	<0.001***	0.646
Generic	*Head*	*Ang. Vel.*	*Entropy*	*Entropy*	73	<0.001***	−0.548
Expert	*Trunk*	*Ang. Vel.*	*Static Periods*	*Ratio (%)*	73	<0.001***	0.524
	Total Body	*Velocity*	*Static Periods*	*Std. Duration (s)*	74	<0.001***	0.834
				Max. Duration (s)	40	<0.001***	0.835
	Head	*Ang. Vel.*	*Static Periods*	*Mean Duration (s)*	76	0.001**	0.857
	T8	*Ang. Vel.*	*Static Periods*	*Counts per Minute*	79	0.001**	−0.601
	Upper Extremities	*Velocity*	*Static Periods*	*Std. Duration (s)*	81	0.002**	0.780
Generic	*Head*	*Ang. Vel.*	*Mean*	*Mean*	82	0.002**	−0.475
Expert	*Head*	*Ang. Vel.*	*Static Periods*	*Max. Duration (s)*	85	0.003**	0.637
	Upper Extremities	*Velocity*	*Static Periods*	*Ratio (%)*	87	0.003**	0.553
	Trunk	*Ang. Vel.*	*Static Periods*	*Max. Duration (s)*	89.500	0.004**	0.692
		Velocity	*Static Periods*	*Max. Duration (s)*	89.500	0.004**	0.664
	Left Hand & Right Hand	*Ang. Vel.*	*Static Periods*	*Max. Duration (s)*	90.500	0.004**	0.408
	Total Body	*Velocity*	*Static Periods*	*Ratio (%)*	91	0.004**	0.528
	Head	*Ang. Vel.*	*Static Periods*	*Std. Duration (s)*	92	0.005**	0.776
	Trunk	*Velocity*	*Static Periods*	*Std. Duration (s)*	92	0.005**	0.672
	Upper Extremities	*Velocity*	*Static Periods*	*Max. Duration (s)*	63	0.005**	0.821
				Mean Duration (s)	94	0.006**	0.660
	Head	*Velocity*	*Static Periods*	*Max. Duration (s)*	95	0.006**	0.742
	Total Body	*Velocity*	*Static Periods*	*Mean Duration (s)*	96	0.007**	0.734
	Left Hand & Right Hand	*Velocity*	*Static Periods*	*Mean Duration (s)*	102	0.011*	0.589
				Std. Duration (s)	104	0.013*	0.617
				Max. Duration (s)	104	0.013*	0.614
	Upper Extremities	*Ang. Vel.*	*Static Periods*	*Max. Duration (s)*	81	0.016*	0.631
Generic	*Head*	*Acc.*	*Mean*	*Mean*	107	0.016*	−0.478
Expert	*Head*	*Velocity*	*Static Periods*	*Std. Duration (s)*	107	0.016*	0.669
	Left Hand & Right Hand	*Velocity*	*Static Periods*	*Ratio (%)*	108	0.018*	0.521
	T8	*Ang. Vel.*	*Static Periods*	*Mean Duration (s)*	119	0.040*	0.510
	Total Body	*Ang. Vel.*	*Static Periods*	*Counts per Minute*	120	0.043*	0.453

Supplementary Table S5.2: Features per Phase

Results of statistical tests of extracted body posture and movement features between TSST and f-TSST from the *Main Study*, computed seperately over *Interview* and *Mental Arithmetics* phases of the (f-)TSST. p-values are corrected for multiple comparisons using the Bonferroni method. *Note*: Only motion features with statistically significant ($p < 0.05$) differences are shown.

Phase	*Feature Type*	*Body Part*	*Channel*	*Type*	*Metric*	W	p	Hedges' g
Mental Arithmetics	*Generic*	*Head*	*Ang. Vel.*	*Entropy*	*Entropy*	51	$<0.001^{***}$	−0.722
Interview	*Expert*	*Trunk*	*Ang. Vel.*	*Static Periods*	*Max. Duration (s)*	68	$<0.001^{***}$	0.873
		Upper Extremities	*Ang. Vel.*	*Static Periods*	*Std. Duration (s)*	68	$<0.001^{***}$	0.770
		Left Hand & Right Hand	*Ang. Vel.*	*Static Periods*	*Std. Duration (s)*	72	0.001^{**}	0.838
					Max. Duration (s)	77	0.002^{**}	0.837
		Upper Extremities	*Velocity*	*Static Periods*	*Mean Duration (s)*	82	0.004^{**}	0.654
		Left Hand & Right Hand	*Velocity*	*Static Periods*	*Mean Duration (s)*	88	0.007^{**}	0.620
			Ang. Vel.	*Static Periods*	*Mean Duration (s)*	88	0.007^{**}	0.754
		Trunk	*Ang. Vel.*	*Static Periods*	*Std. Duration (s)*	89	0.007^{**}	0.770
		Upper Extremities	*Ang. Vel.*	*Static Periods*	*Max. Duration (s)*	53.500	0.008^{**}	0.763
Mental Arithmetics	*Generic*	*Head*	*Ang. Vel.*	*CoV*	*CoV*	93	0.010^{*}	0.646
Interview	*Expert*	*Upper Extremities*	*Ang. Vel.*	*Static Periods*	*Mean Duration (s)*	93	0.010^{*}	0.716
Mental Arithmetics	*Expert*	*Total Body*	*Ang. Vel.*	*Static Periods*	*Max. Duration (s)*	95	0.012^{*}	0.703
		Upper Extremities	*Ang. Vel.*	*Static Periods*	*Ratio (%)*	97	0.014^{*}	0.569
		Head	*Ang. Vel.*	*Static Periods*	*Ratio (%)*	97	0.014^{*}	0.648
Interview	*Expert*	*Total Body*	*Ang. Vel.*	*Static Periods*	*Max. Duration (s)*	99	0.017^{*}	0.789
Mental Arithmetics	*Expert*	*Total Body*	*Ang. Vel.*	*Static Periods*	*Ratio (%)*	102	0.022^{*}	0.653
	Generic	*Head*	*Ang. Vel.*	*Mean*	*Mean*	102	0.022^{*}	−0.522
	Expert	*Upper Extremities*	*Ang. Vel.*	*Static Periods*	*Max. Duration (s)*	104.500	0.028^{*}	0.574
Interview	*Expert*	*Head*	*Ang. Vel.*	*Static Periods*	*Std. Duration (s)*	105	0.028^{*}	0.977
		Trunk	*Velocity*	*Static Periods*	*Max. Duration (s)*	106	0.030^{*}	0.956
		Head	*Ang. Vel.*	*Static Periods*	*Max. Duration (s)*	106.500	0.032^{*}	0.879
Mental Arithmetics	*Expert*	*T8*	*Ang. Vel.*	*Static Periods*	*Counts per Minute*	108	0.035^{*}	−0.535
		Head	*Ang. Vel.*	*Static Periods*	*Max. Duration (s)*	108	0.035^{*}	0.471
		Total Body	*Ang. Vel.*	*Static Periods*	*Mean Duration (s)*	109	0.038^{*}	0.677

Supplementary Table S6 Classification Results – *Main Study*

Features over entire (f-)TSST

Main Study: Mean ± standard deviation of classification performance metrics over the 5-fold model evaluation CV, trained on features extracted over the complete (f-)TSST, respectively. For each evaluated classifier, the classification pipeline combination with the highest mean accuracy is shown. The classification pipelines scoring the highest metrics are highlighted in **bold**.

Scaler	Feature Selection	Classifier	Accuracy [%]	F1-score [%]	Precision [%]
Standard	SFM	RF	**71.6 ± 5.9**	**71.2 ± 4.2**	75.1 ± 13.1
Min-Max	RFE	kNN	67.0 ± 8.4	64.9 ± 11.2	68.1 ± 8.5
Min-Max	SkB	MLP	66.8 ± 6.7	62.4 ± 7.5	**75.4 ± 14.8**
Min-Max	RFE	DT	65.2 ± 9.2	65.9 ± 7.7	66.3 ± 11.3
Min-Max	SkB	NB	64.1 ± 10.0	57.3 ± 12.2	70.3 ± 13.3
Standard	SkB	Ada	63.0 ± 9.3	61.9 ± 7.3	66.3 ± 13.2
Min-Max	SkB	SVM	62.9 ± 5.6	53.8 ± 11.9	69.7 ± 7.0

References

[1] R. Richer, V. Koch, A. Küderle, N. Rohleder, and B. Eskofier, “Dataset "Effect of acute psychosocial stress on body posture and movements" – Pilot Study.” https://osf.io/qvzdg, Feb. 2023.

[2] R. Richer, L. Abel, F. Hauck, M. Kurz, V. Ringgold, A. Küderle, L. Schindler-Gmelch, B. M. Eskofier, and N. Rohleder, “Dataset "Effect of acute psychosocial stress on body posture and movements" – Main Study.” https://osf.io/va6t3/, Feb. 2024.

[3] R. Richer, V. Koch, L. Abel, F. Hauck, M. Kurz, V. Ringgold, V. Müller, A. Küderle, L. Schindler-Gmelch, B. M. Eskofier, and N. Rohleder, “Stresspose-analysis - GitHub.” https://github.com/empkins/stresspose-analysis, 2023.
